# COVID-19: Hands, face, space, fresh air ... and exercise! The missing intervention to reduce disease burden

**DOI:** 10.3399/BJGPO.2021.0088

**Published:** 2021-09-15

**Authors:** Aessa Tumi, Hassan Khan, Sidra Awan, Kosta Ikonomou, Katija Ali, Kristina Frain, Irfan Ahmed

**Affiliations:** 1 St Mary’s Hospital GP VTS scheme, London, UK; 2 University College London, London, UK; 3 University College London Hospital, London, UK; 4 TELUS Health, Vancouver, Canada; 5 Herne Hill Group Practice, London, UK; 6 Barts And The London School of Medicine & Dentistry, London, UK

**Keywords:** COVID-19, Physical activity, exercise, public health, pre-habilitation, primary healthcare

## Introduction

The COVID-19 pandemic has had a significant impact on patient lifestyles with new measures designed to reduce transmission of the virus drastically transforming life as we know it. Public health interventions — alongside advances in medical treatments, vaccine technology, and gene sequencing — have drastically reduced the impact of the pandemic across the world. Yet there has been relatively little focus on the potential role of physical activity (PA) in reducing disease burden during the pandemic. We discuss the latest evidence related to the role of exercise or physical pre-rehabilitation before infection and consider whether this may be an overlooked public health strategy.

### Living with COVID-19: ’Exercise is medicine.’

As we progress through the second year of the pandemic, there has been a renewed focus on adjustments that individuals and society will have to make as we continually adapt to life with COVID-19. Key public health messages regarding the importance of *’* *hands, face, space* *and* *fresh air* *‘* appear prominently on all government briefings but exercise, once famously quoted as being the *‘* *miracle cure* *’* by the Academy of Medical Royal Colleges, is conspicuously absent.^1^ Despite the evidence supporting the physical and mental health benefits of exercise at a population level, there remains a void of information regarding how people can engage in PA safely during the pandemic and a lack specific targeted PA interventions for high-risk patient cohorts.

During the last year, social distancing measures, home working practices, and restricted access to leisure and exercise facilities have had a major impact on individuals' opportunities to stay physically active. Several studies have reported that loneliness, physical inactivity, and weight gain have increased during the COVID-19 pandemic.^2,3^ Physical deconditioning and low exercise capacity (cardiorespiratory fitness) are also associated with many adverse health outcomes such as premature death and increased cardiometabolic disease risk in adult and paediatric populations.^4–7^ Considering this, it has been proposed that structured interventions supporting PA may help to improve health outcomes for all patients and ameliorate the potential negative consequences of lockdown measures.^4^

### What do we know about exercise and COVID-19 so far?

The presence of pre-existing cardiometabolic diseases, obesity, and lower PA levels have all been associated with worsening COVID-19 outcomes; a recent study reported that physically inactive patients are at increased risk of hospitalisation (odds ratio 2.26) compared to those meeting PA guidelines.^8,9^ The proposed benefits of PA are two-fold: acutely reducing hospitalisation and mortality, while reducing the risk of developing long-term cardiometabolic consequences (diabetes, obesity, and cardiovascular disease). Furthermore, widening access to PA may reduce inequalities in COVID-19 outcomes observed in populations at higher risk from physical inactivity and metabolic disease, namely those from Black, Asian, and minority ethnic (BAME) groups.^10^

### Could exercise be used to pre-habilitate patients before infection?

It is predicted that COVID-19 is likely to have a significant burden on healthcare systems worldwide for many years to come, with the prospect of future pandemics. Any efforts to physically pre-habilitate people are likely to be of benefit. In the first instance, PA, — alongside interventions to promote healthy diets, working, and educational policies — could reverse the trend of rising rates of obesity. Exercise can also improve patients’ immune function, metabolic profile, and cardiorespiratory fitness, raising their baseline physiological reserve and ability to respond to acute infection.^4,11^ A recent meta-analysis has also reported that regular exercise is associated with a higher antibody response to vaccinations and highlighted a potential link between regular PA and improved adaptive immunity to infectious diseases.^11^

There are currently several evidence-based clinical care pathways (for example, pulmonary rehabilitation, cardiac rehabilitation, and pre-operative rehabilitation) where rehabilitation and structured exercise have been shown to reduce disease severity, reduce healthcare utilisation, and improve patients’ quality of life.^4,12,13^ The role of exercise for health benefits in the general population is highlighted in the UK Chief Medical Officers’ and World Health Organization guidelines (Box 1), which recommend at least 150 minutes of moderate intensity PA or 75 minutes of vigorous intensity PA, or a combination of the two, alongside resistance training twice per week.^14^

Box 1 **2020 World Health Organization physical activity guidelines for adults aged 18–64 years^18^**

It is recommended that:

- All adults should partake in regular physical activity.
- Adults should do at least 150–300 minute of moderate-intensity aerobic physical activity or 75 min–50 minutes of vigorous intensity of aerobic physical activity, or a combination of moderate and vigorous intensity activity throughout the week for an increase in health benefits.
- Adults should also do muscle-strengthening activities at moderate or greater intensity that involve all major muscle groups on 2 or more days a week, as these provide additional health benefits.

Sedentary behaviour:

- Adults should limit the amount of time spent being sedentary. Replacing sedentary time with physical activity of any intensity (including light intensity).

Evidence-based PA promotion programmes, when integrated into local sports or community facilities, have been shown to increase participation in exercise and encourage positive behaviours change.^15,16^A greater awareness of these programmes could help to promote PA in high-risk groups, or be adapted to pre-habilitate patients to provide similar benefits during the pandemic. Sustained efforts to promote PA must be initiated across society so that interventions go beyond the niche of specialised secondary care pathways and integrate into primary care. Access to remote or telehealth-based exercise initiatives, alongside social prescribing and active policies in work and school environments, may help patients to become more active.^17^

## Conclusion

Low levels of physical activity and cardiometabolic risk factors are associated with worsening health outcomes from COVID-19. Public health initiatives designed to promote PA and reverse the trends of rising physical inactivity and obesity levels in the population could improve outcomes for individuals during the pandemic. In order for this to be successful, PA interventions based upon existing rehabilitation programmes will need to be repurposed and delivered at scale to help all patients stay active during the pandemic.
